# Navigating Bariatric Surgery: Understanding and Managing Short-Term and Long-Term Complications

**DOI:** 10.7759/cureus.48580

**Published:** 2023-11-09

**Authors:** Tamar Tsenteradze, Faris Fayyaz, Chukwuyem Ekhator, Imtiaz Ahmed, Sergio Rodrigo Oliveira Souza Lima, Omar A Daher, Danyal Bakht, Hareem Arif, Sophia B Bellegarde, Nabila N Anika, Faisal F Al-Shaikhly, Azlaan Hussain

**Affiliations:** 1 Medicine and Surgery, Tbilisi State Medical Univerity, Tbilisi, GEO; 2 Surgery, Dow University of Health Sciences, Karachi, PAK; 3 Neuro-Oncology, New York Institute of Technology, College of Osteopathic Medicine, Old Westbury, USA; 4 Medicine and Surgery, Jinnah Medical & Dental College, Jinnah Sindh Medical University, Karachi, PAK; 5 Plastic Surgery, Hospital da Bahia, Salvador, BRA; 6 Obstetrics and Gynaecology, Beirut Arab University, Tripoli, LBN; 7 Medicine and Surgery, Mayo Hospital, Lahore, PAK; 8 Internal Medicine, Ghulam Muhammad Mahar Medical College, Sukkur, PAK; 9 Pathology and Laboratory Medicine, American University of Antigua, Coolidge, ATG; 10 Medicine and Surgery, Holy Family Red Crescent Medical College Hospital, Dhaka, BGD; 11 Medicine and Surgery, University of Jordan, Amman, JOR

**Keywords:** marginal ulcers, infections, hemorrhages, strictures, anastomotic leaks, long-term complications, short-term complications, weight loss surgery, obesity, bariatric surgery

## Abstract

Obesity is a global public health crisis associated with numerous medical conditions and increased mortality rates. Weight loss surgery, or bariatric surgery, has become a crucial treatment option for clinically severe obesity. Bariatric surgery is an effective treatment for severe obesity but it carries the potential for various complications, both in the short and long term. This article provides a comprehensive overview of these complications, aiding healthcare professionals in their management and patients in understanding the risks associated with bariatric surgery. The review explores the short-term complications of bariatric surgery, emphasizing anastomotic leaks, strictures, hemorrhages, infections, marginal ulcers, gastroesophageal reflux disease (GERD), and dumping syndrome. It provides insights into the diagnosis and management of these complications, emphasizing the importance of early recognition and intervention. Furthermore, the article delves into the late complications of adjustable gastric banding (LAGB), vertical sleeve gastrectomy (VSG), Roux-en-Y gastric bypass (RYGB), and biliopancreatic diversion with duodenal switch (BPDDS). It discusses complications such as band slippage and erosion in LAGB, nutritional deficiencies in VSG and RYGB, and unique complications related to BPDDS.

## Introduction and background

The obesity epidemic has reached alarming proportions globally, making it one of the most pressing public health concerns of our time [1]. As obesity rates have increased, so has the need for efficient therapies to address this intricate and widespread issue. Multiple studies have established a strong association between obesity and mortality. An extensive prospective cohort investigating the association between BMI and mortality determined that a higher BMI was associated with an increased risk of death, particularly among non-smokers and people aged ≥ 50 years [2]. Numerous medical conditions such as diabetes, nonalcoholic fatty liver disease, gallbladder disease, cardiovascular disease, hypertension, dyslipidemia, endocrine changes, musculoskeletal disorders, cancer, and pulmonary complications have been linked to obesity. Additionally, obesity has a detrimental influence on psychological functioning and health-related quality of life and is linked to increased rates of stigmatization and discrimination.

The compelling evidence linking obesity to mortality and various medical conditions has driven the demand for effective treatments. Weight loss surgery, commonly known as bariatric surgery, has become a popular and highly successful treatment option for those with clinically severe obesity [3,4]. It not only significantly reduces weight but also has the ability to lessen or even eliminate comorbidities associated with obesity. The rising severity of the obesity pandemic is reflected in the increase in demand for bariatric surgery.

Although behavioral and pharmaceutical therapies for obesity may result in a short-term weight loss of around 5-10% of body weight, their long-term effectiveness is still restricted [5]. Following these therapies, weight return frequently happens between six and twenty-four months later, along with a decline in health-related gains. On the contrary, bariatric surgery can result in significant and long-lasting weight loss, anywhere between 50% and 75% of extra body weight, with some studies showing weight maintenance up to 16 years after surgery [6-8]. Bariatric surgery is now recognized as the most effective and long-lasting therapy for clinically severe obesity as a result of its excellent effectiveness. The popularity of bariatric surgery is rising for a variety of reasons. The development of laparoscopic procedures, for example, has increased safety and resulted in shorter hospital stays [9]. The continuous rise of bariatric surgery procedures has also been significantly influenced by increased awareness among patients and physicians, media attention highlighting celebrity patients' experiences, and extended coverage by health insurance companies and third-party payers. In this review, we searched the literature for articles that discussed complications associated with various types of bariatric surgery and compared and discussed the findings.

## Review

Types of bariatric surgery

Bariatric surgical procedures can be classified into three main categories based on their functions: restrictive, combined (restrictive and malabsorptive), and primarily malabsorptive. These procedures aim to achieve weight loss through different mechanisms. Restrictive procedures include laparoscopic adjustable gastric banding (LAGB), vertical banded gastroplasty (VBG), and laparoscopic sleeve gastrectomy (LSG). LAGB involves the laparoscopic insertion of a silicone gastric band at the upper end of the stomach, creating a small proximal gastric pouch with a volume of approximately 30 ml [10]. To reach the desired amount of constriction, the band is inflated with a saline solution, leading to early satiety and moderate weight loss. The degree of inflation is tailored to each individual. In order to induce an early feeling of fullness, the stomach is stapled from front to back, below the gastroesophageal junction, and the exit stoma is constrained with a polypropylene band in VBG [11]. However, VBG is no longer widely used because of its unexpectedly high complication rate. LSG develops a stomach "sleeve" that is approximately 60-120 mL in size and extends from the esophagus to the duodenum. In this surgical procedure, almost 80% of the stomach is removed, which lowers the amount of the hunger-related hormone ghrelin produced, reducing appetite and enhancing fullness [12].

Malabsorptive procedures include jejunoileal bypass. A major piece of the absorptive loop is effectively removed during this treatment by joining the upper small intestine to the distal small intestine, leaving just around 35 cm of healthy absorptive small intestine. Although it works well for losing weight, there is a considerable risk of mortality, severe starvation, and organ failure. It is no longer suggested as a result of these dangers [13,14]. Combined restrictive and malabsorptive procedures include Roux-en-Y gastric bypass (RYGB) and biliopancreatic diversion with duodenal switch (BPDDS). Gastric bypass has evolved significantly since its inception in the late 1970s. It first involved establishing a small gastric pouch by horizontally dividing the upper stomach. Later, a gastrojejunostomy was performed to restore gastrointestinal continuity. The stomach is now primarily divided, as opposed to partitioned, and the size of the gastric pouch has been steadily reduced. The invention of devices that simultaneously staple and split the stomach has made this alteration easier. The Roux-en-Y limb also referred to as the biliopancreatic limb, alimentary limb, or common channel, is made up of different lengths of the small intestine. While RYGB is mostly restrictive, some malabsorption is also brought on by the fact that parts of the stomach, duodenum, and upper jejunum are bypassed [15,16]. A prosthetic band like the one used in VBG can be added to the gastric pouch to address the risk of weight gain after gastric bypass surgery. However, this method has risks similar to VBG, such as band-related complications. BPDDS combines stomach reduction with alterations to the small intestine's path. Even though it causes significant weight reduction, it is linked to greater risks of complications, such as malabsorption, protein malnutrition, vitamin and mineral deficiencies, anemia, osteoporosis, and anastomotic ulceration [13].

Most of bariatric operations now use laparoscopic techniques as their preferred technique since they result in fewer postoperative complications, shorter hospital stays, and shorter recovery periods than open procedures [9,17]. The choice of surgical method, however, is influenced by a number of variables, including the patient's anatomy and the surgeon's background. Revisional surgery could be required by individuals who fail to lose enough weight or have issues following their original bariatric procedure. Resolving those issues might require altering or changing existing procedures or carrying out a different kind of surgery. In the short term, bariatric surgery typically leads to significant weight loss and improvements in obesity-related comorbidities, including diabetes, hypertension, dyslipidemia, sleep apnea, and more. Following bariatric surgery, long-term success is dependent on variables like diet compliance, exercise, and postoperative monitoring. Patients frequently report a higher quality of life and a lower risk of illnesses linked to fat.

Short-term complications

In the fight against obesity, bariatric surgery is a potent weapon that frequently produces considerable weight reduction and better health results. However, it entails potential consequences, some of which can be serious and even life-threatening if not treated right away, just like any surgical procedure. This section delves into the early complications of bariatric surgery, with a focus on procedures such as sleeve gastrectomy (SG), RYGB, VSG, and BPDDS.

Anastomotic leaks have the potential to drastically raise morbidity and death rates, making them possibly the most worrisome complication of any bariatric surgery [18]. These leaks happen when the anastomotic seal fails, allowing intestinal or stomach contents to flow into the abdominal cavity or other surrounding structures. Gastrojejunal anastomosis (GJA), gastrobronchial fistulae, and even gastroenteric fistulae are among the sites where leaks can develop [19-21]. Following bariatric surgery, there is a higher chance of leakage due to a number of risk factors as follows: (i) Revisional Surgery: Leaks are more likely to occur in patients who have had revisional bariatric surgery; (ii) High BMI: Patients who have a BMI of more than 50 kg/m^2^ are more likely to have leaks [19-21]; (iii) Dysmetabolic Syndrome X: Patients with this constellation of metabolic complications are likewise more prone to leaks [20].

Patients with postoperative leaks commonly experience a particular set of symptoms such as persistent tachycardia, dyspnea, fever, and discomfort or pain in the abdomen. Three days following surgery is the typical period for leak symptoms to appear [22]. It is important to take into account that these symptoms might occur after the patient has been discharged from the hospital, prompting a visit to the emergency department. For patients with suspected leaks, a thorough diagnostic evaluation is essential. Typically, this assessment includes the following: (i) Abdominal CT scan: A useful method for finding leaks is an abdominal CT scan with oral contrast. Even in cases where there is no contrast extravasation, CT scans can detect leaks. It is important to note that 60-80% of leaks at the GJA or in an SG are discovered by CT [22,23]; (ii) Upper Gastrointestinal Series (UGS): A UGS may also be used to find leaks at the GJA, but it is less accurate than CT. However, after an RYGB, neither CT nor UGS is useful for excluding a leak at the jejuno-jejunal anastomosis (JJA) [24]; (iii) Surgical Exploration: In hemodynamically unstable patients or those with persistent tachycardia, surgical exploration may be necessary despite negative radiologic studies. During surgery, the priorities include removing contamination, controlling the leak with closed suction drains, and establishing feeding access. Repairing the leak is optional, depending on its feasibility and the patient's condition. It is critical to understand the key distinctions between sleeve leaks and RYGB leakage. Sleeve leaks happen in a high-pressure environment, frequently at the sleeve's topmost point, where the blood supply is weak [25-27]. As opposed to this, RYGB produces a low-pressure gastric pouch, which lowers the incidence of leaks (between 0.6% and 4.4% of patients) [28]. Because of this pressure differential, non-operative management techniques can successfully handle RYGB leaks without sealing or repairing the perforation. Treatment for leaks depends on the type of bariatric surgery and the condition of the patient (Table 1).

**Table 1:** Treatment strategies for anastomotic leak. SG: sleeve gastrectomy, RYGB: Roux-en-Y gastric bypass, GJA: gastrojejunal anastomosis

Intervention	Procedure
Endoluminal Intervention	Image-guided drainage procedures can be used on stable patients who have leakage following an SG. Leak control options include endoluminal intervention and covered stenting. Endoluminal treatments including clip placement, stents, or vacuum dressings might be taken into consideration to assist in stopping persistent RYGB leaks that continue for more than 30 days [25,27].
Nutritional Support	Supportive nutrition is essential for patients with leakage. Total parenteral nutrition should be avoided in favor of enteral feeding distal to the GJA, and multiple sites can be utilized for implanting feeding tubes to guarantee sufficient nutrition [10].
Surgical Repair	Surgery can be required in certain cases to repair the leak. Closing the leak, placing interrupted sutures, or covering the repair site with a modified Graham patch are all surgical options [26].

Stenosis, twisting, or kinking of the gastrointestinal tract can lead to significant complications following bariatric surgery. These conditions may cause regurgitation, difficulty passing food or liquids, and a feeling of trapped food, all of which can contribute to malnutrition and vitamin deficiencies. Stenosis commonly occurs after RYGB, with an incidence ranging from 8% to 19%. It is more prevalent when end-to-end anastomosis staplers are used [29]. Diagnosis is confirmed through a UGS, which shows a failure of contrast to pass through the GJA. The primary therapy is endoscopic balloon dilatation, with a target stenosis diameter of 15 mm [30]. Stenosis following SG occurs less often, with a frequency of 0.7-2%. Endoscopic balloon dilation is commonly used for treating it [31-33]. The mini-gastric bypass and the duodenal switch are more commonly linked to small intestine twists and kinks. The diagnosis might be difficult since symptoms may be intermittent. Surgical intervention is frequently required to address these complications [34].

Infections are a known risk in any surgical procedure, and bariatric surgery is no exception. Due to a number of variables, such as the surgical site itself and comorbidities associated with obesity, patients following weight reduction surgery may be at an elevated risk for infections. One of the most typical forms of infections linked to bariatric surgery is surgical site infection (SSI). These infections may develop in the abdominal cavity or at the site(s) of the incision. Obesity, diabetes, prolonged surgery, and poor glycemic control are risk factors for SSI [35]. An access port is positioned beneath the abdominal skin in individuals who have adjustable gastric bands. At the location of the port, infections might manifest as localized redness, discomfort, or swelling. These infections may require antibiotic treatment and, in some cases, removal of the port [36]. Prevention of infections is a critical component of postoperative care in bariatric surgery. During surgery, surgeons and the surgical team must adhere to stringent aseptic (sterile) procedures. The risk of SSI can be decreased by giving antibiotics prior to surgery. It is recommended to quit smoking before surgery since smokers have a greater risk of infection. For diabetic patients, maintaining optimal glycemic control before and after surgery is crucial [37].

Marginal ulcers are a known complication of RYGB surgery [38]. They are characterized by the development of ulcerations near the gastrojejunostomy. Symptoms of these ulcers include nausea, vomiting, and stomach discomfort. Smoking, nonsteroidal anti-inflammatory drugs (NSAIDs), insufficient blood flow to the anastomosis, heavy alcohol intake, and subpar surgical skills are some risk factors that increase the risk of developing marginal ulcers. Endoscopy is often used to diagnose marginal ulcers because it provides a clear view of the affected region [39]. Proton pump inhibitors (PPIs), quitting smoking, and refraining from NSAIDs and alcohol are other treatment options. The underlying problem may need to be addressed surgically when ulcers are persistent or resistant to medicinal therapy.

Gastroesophageal reflux disease (GERD) is a condition where stomach acid frequently flows back into the esophagus, causing symptoms such as heartburn, regurgitation, and chest pain. While bariatric surgery frequently reduces GERD symptoms, in some people it may continue or worsen [40]. When the high-pressure environment inside the stomach is disturbed following an SG, GERD may develop. A portion of the stomach is removed during SG, which might expose the remaining stomach to more acid. Additionally, the procedure can alter the normal antireflux mechanisms of the lower esophageal sphincter (LES) [41]. Although further testing, such as pH monitoring or endoscopy, may be required to determine the degree of reflux and its effects on the esophagus, the diagnosis is frequently made based solely on symptoms alone [40,42]. The management of GERD following bariatric surgery includes the following: (i) Lifestyle modifications: Patients are advised to make dietary and lifestyle changes, such as avoiding acidic foods, elevating the head of the bed, and losing excess weight; (ii) Medication: Symptoms of GERD can be relieved with over-the-counter antacids, H2-receptor antagonists, and PPIs; (iii) Surgical Revision: A surgical revision of the procedure may be considered in situations with severe or persistent GERD. This may include switching to a RYGB or undergoing further operations that recreate a stronger anti-reflux barrier.

Dumping syndrome develops when hyperosmolar chyme passes through the small intestine too rapidly. The systemic and gastrointestinal symptoms that might result from this fast transit are many. There are two types of dumping syndrome, early and late [43]. Early dumping syndrome manifests as symptoms including nausea, vomiting, stomach cramps, diarrhea, and flushing within 10 to 30 minutes of eating [43]. It is often triggered by the rapid entry of undigested food into the small intestine. Late dumping syndrome typically occurs one to three hours after eating and is characterized by symptoms like sweating, weakness, dizziness, and palpitations [43]. It is brought on by reactive hypoglycemia, which happens when too much insulin is released in reaction to a sudden inflow of glucose in the small intestine. Dumping syndrome most commonly occurs after RYGB due to the rapid emptying of the small gastric pouch into the jejunum. The inherent pyloric valve is bypassed during this procedure, allowing food to pass straight into the small intestine [10]. Managing dumping syndrome involves dietary modifications and medication. Patients are instructed to consume smaller, more frequent meals and avoid diets rich in sugar. Complex carbohydrates and proteins may be better tolerated. In late dumping syndrome, medications such as acarbose or octreotide may be administered to slow down digestion and lower the risk of hypoglycemia [10].

Late complications

Late Complications of LAGB

Adjustable gastric band complications associated with LAGB can vary in severity and timing. Band slippage, band erosion, esophageal dilatation, blockage, and device-related issues are examples of late complications that might develop months to years after the initial surgery.

Band slippage is a relatively common late complication, occurring in approximately 8% of patients who undergo LAGB [44,45]. This happens when one stomach wall or side slides through the band's opening, creating a bigger gastric pouch above the band [36]. Vomiting after meals, either right away or later, a sense of satiety eased by vomiting, and discomfort in the upper abdomen are all indications of band slippage. Diagnosis is made through plain abdominal X-rays. A slipped band is indicated by an irregular "phi angle," one greater than 58°, or by the appearance of an "O sign" (the complete ring of the band) on X-rays [46]. Other radiographic indicators include an air-fluid level above the band and an inferior displacement of the band margin [47]. To alleviate symptoms, the band should first be drained of fluid. Patients may be put on a liquid diet and referred for elective band removal if the band slippage resolves. Emergency surgery for band removal and, in extreme circumstances, excision of ischemic stomach tissue may be necessary if symptoms persist or worsen [34].

Band erosion is an uncommon late complication that affects only a tiny proportion of individuals (2.8%, on average) [48]. The band starts to erode into the stomach wall, which might cause nonspecific symptoms such as upper abdominal discomfort, lack of restriction, melena, or reflux. Upper endoscopy is frequently used to diagnose such cases because it can detect if the band has partially or completely eroded into the stomach. The management of band erosion depends on its extent. Complete or near-complete intraluminal bands can be removed endoscopically [49,50]. Laparoscopic band removal and erosion site repair may be necessary for partial erosions. Dietary restrictions and antibiotics may also be included in the treatment regimen.

Late Complications of VSG

VSG is considered a restrictive bariatric procedure with relatively few complications. However, late complications can still happen, such as marginal ulcers, dietary deficits, and gallstone development [51,52]. Nutritional deficiencies can develop over time as a result of decreased food consumption and poor vitamin absorption. Common dietary deficiencies include those in iron, vitamin D, folic acid, vitamin B1, and vitamin B12. Regular blood tests are required to monitor nutritional status. Patients may require dietary supplements for the rest of their lives to treat these deficiencies. Rapid weight loss after VSG may increase the risk of gallstone development. Ursodeoxycholic acid may be administered to minimize this risk.

Late Complications of RYGB

The combination of restrictive and malabsorptive elements in RYGB makes it both extremely effective and prone to late complications. Patients with RYGB, like those with VSG, are susceptible to dietary deficits, especially those involving iron, vitamin D, vitamin B12, and folate. Nutritional status is often evaluated by blood testing. To overcome deficiencies, lifelong supplementation and monitoring are crucial. Some patients may develop late dumping syndrome, characterized by symptoms such as sweating, dizziness, and diarrhea after consuming high-sugar or high-carbohydrate foods. Symptoms and food history are used to make the diagnosis. Dietary adjustments, such as staying away from meals high in sugar, can help control dumping syndrome. Late complications may include the development of anastomotic strictures (narrowing of the connection between the stomach and intestine) and ulcers [53]. Strictures and ulcers can be identified through upper endoscopy. For strictures, endoscopic dilatation may be required, but for ulcers, medication, and dietary changes could prove essential. At the surgery site, incisional hernias can also develop, needing surgical correction [52-54]. Some patients may experience late weight return, which frequently necessitates extra interventions like switching to a different bariatric operation or lifestyle modifications.

Late Complications of BPDDS

BPDDS is a complex procedure with a higher risk of late complications [55,56]. Patients are at a significant risk of acquiring severe dietary deficiencies, including protein, vitamins, and minerals, due to the extreme malabsorption associated with BPDDS. Frequent monitoring of nutrient levels with supplementation is essential. Similar to RYGB, late dumping syndrome can also occur in BPDDS patients [43]. The diagnosis is made based on the symptoms and dietary history. The main form of treatment is modification of diet. Bowel obstructions may also develop and are identified through imaging like CT scans. Surgical intervention is often necessary to relieve obstructions. Patients with BPDDS are also susceptible to liver conditions, such as non-alcoholic fatty liver disease [57]. The functioning of the liver is tracked through imaging scans and liver function testing. The prevention of liver disease requires careful monitoring, weight control, and dietary modifications in such patients.

## Conclusions

While bariatric surgery offers substantial benefits in addressing obesity and related health concerns, patients and healthcare professionals must be aware of potential complications. The choice of surgery, diligent postoperative care, and lifelong nutritional monitoring are crucial for ensuring the long-term success and safety of bariatric procedures for severe obesity. Short-term complications encompass anastomotic leaks, strictures, hemorrhages, infections, marginal ulcers, GERD, and dumping syndrome. Timely identification and intervention are crucial for effective management, with the choice of treatment dependent on the specific complication and patient's condition. In the long term, different bariatric surgeries entail distinct late complications. LAGB can lead to issues like band slippage, erosion, and esophageal dilatation. VSG may result in marginal ulcers, dietary deficiencies, and gallstones. RYGB can cause dietary deficits, late dumping syndrome, anastomotic strictures, ulcers, hernias, and weight regain. BPDDS poses a risk of severe dietary deficiencies, bowel obstructions, and liver problems.
